# Comparative Transcriptome Analysis Reveals the Process of Ovarian Development and Nutrition Metabolism in Chinese Mitten Crab, *Eriocheir Sinensis*

**DOI:** 10.3389/fgene.2022.910682

**Published:** 2022-05-24

**Authors:** Qiangmei Feng, Meimei Liu, Yongxu Cheng, Xugan Wu

**Affiliations:** ^1^ Centre for Research on Environmental Ecology and Fish Nutrition of Ministry of Agriculture and Rural Affairs, Shanghai Ocean University, Shanghai, China; ^2^ Key Laboratory of Marine Biotechnology of Jiangsu Province, Jiangsu Ocean University, Lianyungang, China; ^3^ Shanghai Engineering Research Center of Aquaculture, Shanghai Ocean University, Shanghai, China; ^4^ National Demonstration Centre for Experimental Fisheries Science Education, Shanghai Ocean University, Shanghai, China

**Keywords:** *Eriocheir sinensis*, ovarian development, hormones, nutrient deposition, comparative transcriptome

## Abstract

Ovarian development is a key physiological process that holds great significance in the reproduction of the Chinese mitten crab (*Eriocheir sinensis*), which is an economically important crab species for aquaculture. However, there is limited knowledge for the regulatory mechanisms of ovarian development. To study the molecular mechanisms of its ovarian development, transcriptome analysis was performed in the ovary and hepatopancreas of *E. sinensis* during ovarian stages I (oogonium proliferation), II (endogenous vitellogenesis), and III (exogenous vitellogenesis). The results showed that 5,520 and 226 genes were differentially expressed in the ovary and hepatopancreas, respectively. For KEGG enrichment analysis, the differentially expressed genes in the ovary were significantly clustered in phototransduction-fly, phagosome, and ECM-receptor interaction. Significantly enriched pathways in the hepatopancreas included fatty acid biosynthesis, fatty acid metabolism, and riboflavin metabolism. Further analysis showed that 25 genes and several pathways were mainly involved in oogenesis, including the ubiquitin-proteasome pathway, cyclic AMP-protein kinase A signaling pathway, and mitogen-activated protein kinase signaling pathway. Twenty-five candidate genes involved in vitellogenesis and endocrine regulation were identified, such as vitellogenin, vitellogenin receptor, estrogen sulfotransferase, ecdysone receptor, prostaglandin reductase 1, hematopoietic prostaglandin D synthase and juvenile hormone acid O-methyltransferase. Fifty-six genes related to nutritional metabolism were identified, such as fatty acid synthase, long-chain-fatty-acid-CoA ligase 4, 1-acyl-sn-glycerol-3-phosphate acyltransferase 4, fatty acid-binding protein, and glycerol-3-phosphate acyltransferase 1. These results highlight the genes involved in ovarian development and nutrition deposition, which enhance our understanding of the regulatory pathways and physiological processes of crustacean ovarian development.

## Introduction

Ovarian development is a complex physiological process in crustacean reproduction (Meusy and Payen, 1988). Female crustaceans usually begin vitellogenesis and ovarian development after the puberty molt (Wang et al., 2014; Wu et al., 2017). Ovarian development can be divided into oogonium proliferation stage, endogenous vitellogenic stage, exogenous vitellogenic stage, and oocyte maturation stage (Wu et al., 2017). Previous studies have shown that the ovary and hepatopancreas are the primary vitellogenesis site in the endogenous and exogenous vitellogenic stages, respectively (Li et al., 2006). During crustacean ovarian development, large amounts of nutrients in the hepatopancreas would be transported to developing oocytes, especially vitellin (Vn) and lipids (Cheng et al., 1998). Previous studies have revealed that vitellogenin (Vg) synthesized in the hepatopancreas would be transported to oocytes by vitellogenin receptor (VgR) synthesized in the ovary (Bai et al., 2016; Guo et al., 2019). However, the physiological process of nutrient metabolism and deposition, including digestion, absorption, transportation, storage, anabolism, and catabolism of nutrients, are highly complicated during the ovarian maturation cycle of crustaceans (Vogt, 1994). To date, gene expression patterns related to nutritional metabolism during ovarian maturation remain unknown.

Various hormonal factors have been employed to positively control ovarian development in crustaceans (Subramoniam, 2000; Nagaraju, 2011). These hormonal factors originating from different endocrine organs are involved in the ovarian development of crustaceans, either individually or in synergy with one another. These organs mainly include the brain ganglion, eyestalk, Y-organ (YO), and mandibular organ (MO), which jointly regulate ovarian development of crustaceans by secreting hormones such as peptides, biogenic amines, steroid hormones, prostaglandins, and methyl farnesoate (MF) (Nagaraju, 2011). The main targets of these hormones are the ovaries and hepatopancreas. Endocrine hormones have complex regulatory pathways involved in ovarian development in crustaceans. For example, ecdysteroids can promote vitellogenesis of crustacean (Gong et al., 2015), whereas MF regulates the vitellin synthesis pathway (Homola and Chang, 1997). However, the regulatory mechanism underlying this phenomenon remains unclear.

The Chinese mitten crab *Eriocheir sinensis* is an economically important aquaculture species favored by consumers due to their delicacy (China Fisheries Yearbook, 2021). The status of ovarian development directly affects the quality and subsequent reproduction of adult *E. sinensis* and cannot be controlled artificially (Wu et al., 2017). Therefore, it is crucial to understand the mechanism of ovarian development in *E. sinensis*. The transcriptome can be used to mine gene expression patterns, obtain abundant genetic resources, and initially understand physiological processes. Advances in transcriptome technology have greatly improved our ability to analyze non-model species (Mardis, 2008). In the present study, changes in genes in the ovary and hepatopancreas were identified during the ovarian development of *E. sinensis* by transcriptomic analysis. The results of this study not only reveal the genes involved in ovarian development, nutrition transport and accumulation, but will also enhance the understanding of molecular regulation pathways and physiological processes of ovarian development in crustaceans.

## Materials and Methods

### Sample Collection

Between July 2015 and November 2015, healthy and intact female crabs (body weights of 70–135 g) were obtained monthly from the experimental ponds of the Chongming Research Base of Shanghai Ocean University. The crabs were maintained in aquarium tanks and acclimated for at least 7 days before sacrifice, as described previously (Wu et al., 2014). Based on the study of Wu et al. (2017), the ovarian development of *E. sinensis* was classified into five stages. Specifically, the stage I ovary was small and the dominant gametocyte were oogonia and previtellogenic oocytes. The dominant type of gametocytes in stage II ovary was endogenous vitellogenic oocytes. The dominant type of gametocytes in stage III ovary was exogenous vitellogenic oocyte. The dominant type of gametocytes in stage IV ovary was nearly mature oocytes. The stage V ovary was filled with mature oocytes (Table 1). Transcriptome analysis was performed on individuals at different ovarian stages, namely, the oogonium proliferation stage (Stage I), endogenous vitellogenic stage (Stage II), and exogenous vitellogenic stage (Stage III). Three individual crabs at each stage were sampled and anesthetized on ice, and then the ovaries and hepatopancreas were removed from the crabs for transcriptome analysis. Three biological replicates were performed per tissue at each stage for a total of nine crabs.

**TABLE 1:** The main features of the different development stages during the ovarian maturation of *E. sinensis*.

Ovarian Stage	Ovarian Features
Stage I	The ovary is small at oogonium proliferation stage, and the dominant gametocyte at this stage were oogonia and previtellogenic oocytes
Stage II	The ovary appeared milk white or buff, and the dominant type of gametocytes was endogenous vitellogenic oocytes
Stage III	The dominant type of gametocytes at this stage was exogenous vitellogenic oocyte that was surrounded by follicle cells, and the yolk granule was present in the oocyte
Stage IV	Ovary lobes partially covered the branchia, and the dominant type of gametocytes was nearly mature oocytes
Stage V	The ovary appeared deep purple, and the ovary is filled with mature oocytes, the yolk granule was lager and distributed evenly in the cytoplasm of mature oocytes

### RNA Extraction and Transcriptomic Sequencing

Total RNA was isolated from each tissue using TRIzol reagent (Invitrogen, USA). RNA library construction and sequencing were performed as previously described (Xu et al., 2020). RNA purity, concentration, and integrity were determined using a NanoPhotometer spectrophotometer (IMPLEN, CA, USA), Qubit 2.0 Fluorometer (Life Technologies, CA, USA), and Agilent 2,100 Bioanalyzer (Agilent Technologies, CA, USA), respectively. Next, the NEBNext Ultra RNA Library Prep Kit for Illumina platform was used to generate sequencing libraries according to the manufacturer’s instructions. mRNA was purified from total RNA using poly-T oligo-attached magnetic beads, and then mRNA was randomly fragmented in fragmentation buffer. The mRNA fragments were used as templates to synthesize double-stranded cDNA, which was further purified with the AMPure XP system (Beckman Coulter, Beverly, USA). Next, the ends of the double-stranded cDNA fragments were repaired using poly (A) and adapter ligation. The cDNA fragment size was selected using the AMPure XP system (Beckman Coulter, Beverly, USA) to obtain fragments preferentially 250–300 bp in length. Sequencing libraries were created after PCR amplification and sequenced using an Illumina HiSeq2500 platform.

### Transcriptome Assembly

The original image data were transferred to raw sequence data and saved as FASTQ files. Clean reads were obtained by removing those with adapters, ploy-N reads, and low-quality reads from the raw sequence data. All subsequent analyzes were based on high-quality clean data. Clean reads were aligned to the reference genome assembly using HISAT v2.0.4 program. HTSeq v0.6.1 program was used to count the read numbers mapped to each gene. Finally, gene expression levels were normalized as FPKM (expected number of Fragments Per Kilobase of transcript sequence per Millions base pairs sequenced). Raw data were uploaded to the National Center for Biotechnology Information (NCBI) (accession number is PRJNA660118).

### Differential Expression Analysis and Functional Annotation

Differential expression analysis was performed using the DESeq package (1.10.1) (Anders and Huber, 2010). The resulting *p*-values were adjusted using Benjamini and Hochberg corrections to control the false discovery rate. Genes with an adjusted *p*-value (*P*_adj_) < 0.05 were assigned as differentially expressed.

In this study, GO enrichment analysis of differentially expressed genes (DEGs) was performed using GOseq (Young et al., 2010). GO terms with corrected *p*-value < 0.05 were considered significantly enriched by DEGs. The KEGG database was used to further interpret the functions of the genes. The statistical enrichment of DEGs in KEGG pathways was performed using KOBAS software (Mao et al., 2005).

### Validation of DEGs by Quantitative Real-Time PCR

Eight genes involved in ovarian development and triacylglycerol (TAG) metabolism were randomly selected for validation using quantitative real-time PCR (qRT-PCR). A reverse first strand cDNA synthesis kit (RR036A, Takara Bio, Japan) was used to synthesize first-strand cDNA. Primer pairs were designed using Primer 6.0, and all the primer sequences are listed in Supplementary Table S1. β-actin was used as an internal control to normalize target gene expression. qRT-PCR was performed in a FAST-7500 system (ABI-7500, Thermo Fisher, Singapore) using a TB-green ^®^ Premix Ex TaqTM II kit (RR420A, Takara Bio, Japan). The reaction system and PCR protocols were performed as previously described (Pan et al., 2018). Each ovarian stage had three replicate crabs for each gene, and the relative expression levels were calculated using the 2^−ΔΔCt^ method (Livak and Schmittgen, 2001). The qRT-PCR results were compared to the FPKM value of each stage in RNA-seq for RNA-Seq validation.

### Statistical Analysis

Statistical analyzes were performed using SPSS Statistics V22.0 (IBM Corporation, NY, USA). The data are presented as the mean for each ovarian stage, and homogeneity of data variance was evaluated by Levene’s test. The Student’s t-test was used to determine significant differences between the two ovarian stages. When homogeneity of variances was not achieved, the data were subjected to Welch’s t-test with Bonferroni correction. Statistical significance was set at *p* < 0.05.

## Result

### Sequencing and *De Novo* Assembly

After adaptor sequences and low-quality reads were removed, a total of 360 million clean reads were obtained from the three stages of the *E. sinensis* transcriptome, including 60,823,878 (Stage I), 62,784,261 (Stage II), and 59,423,899 (Stage III) reads from the ovary libraries; and 57,692,985 (Stage I), 61,218,585 (Stage II), and 58,604,286 (Stage III) reads from the hepatopancreas libraries (Table 2).

**TABLE 2:** Raw reads and quality control of reads for cDNA libraries of female *E. sinensis*.

Tissue	Stage	Raw Reads	Clean Reads	Q20 (%)	Q30 (%)	GC Content (%)
Ovary						
	Stage I	61717725	60823878	96.96	92.63	51.81
	Stage II	64145415	62784261	97.04	92.80	52.16
	Stage III	60738194	59423899	97.01	92.74	51.74
Hepatopancreas						
	Stage I	58854117	57692985	97.06	92.79	52.13
	Stage II	62202730	61218585	96.83	92.41	50.24
	Stage III	59454474	58604286	97.10	92.96	48.99

### Identification of DEGs

**FIGURE 1:**
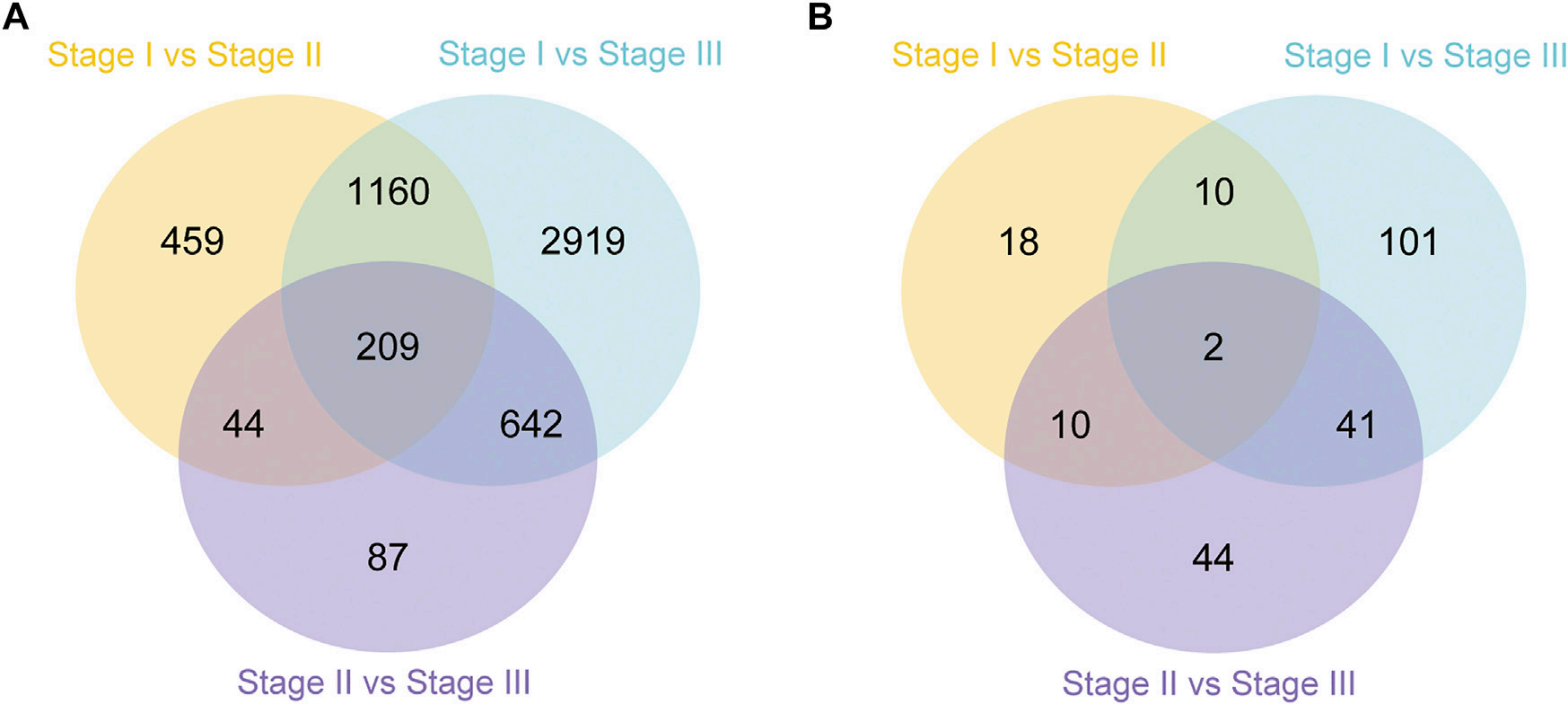
Summary of differentially expressed genes (DEGs) in transcriptome of ovary **(A)** and hepatopancreas **(B)** from different ovarian development stage of *E. sinensis*.

**FIGURE 2:**
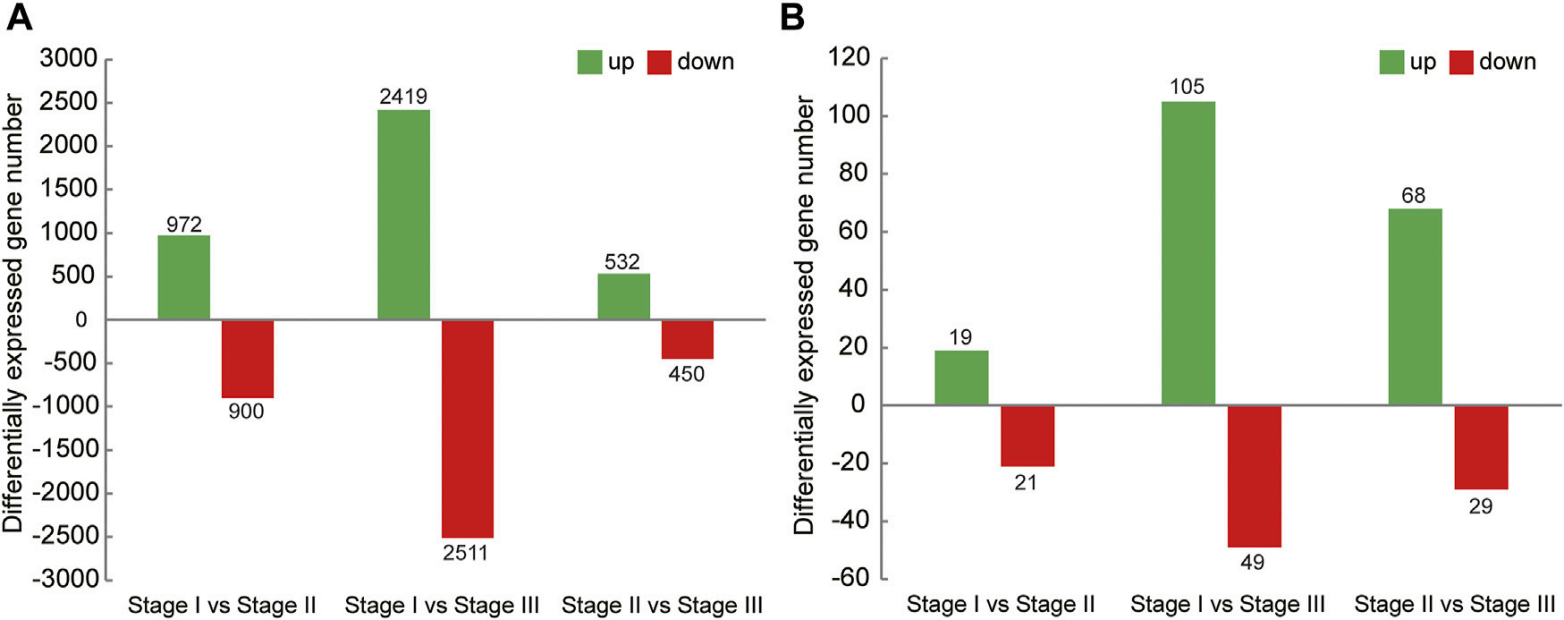
Summary of upregulated and downregulated genes in transcriptome of ovary **(A)** and hepatopancreas **(B)** from different ovarian development stage of *E. sinensis*.

In this study, the analysis of ovary transcriptome data revealed that 5,520 DEGs were annotated successfully across the three stages, of which 209 DEGs were annotated in all stages (Figure 1A). By comparing the databases of stages I and II, 1,872 DEGs were identified, of which 972 and 900 DEGs were upregulated and downregulated, respectively. The comparison results between stages I and III showed that 4,930 DEGs were identified, of which 2,419 and 2,511 DEGs were upregulated and downregulated, respectively. Moreover, the comparison results between stages II and III identified 532 upregulated and 450 downregulated genes (Figure 2A).

A total of 226 DEGs were annotated successfully in the hepatopancreas transcriptome data, of which only two DEGs were annotated in all stages (Figure 1B). We found that the DEGs between stages I and II were relatively few, with only 40 genes identified, of which 19 were upregulated and 21 were downregulated. A total of 154 DEGs were identified between stages I and III, of which 105 and 49 genes were upregulated and downregulated, respectively. Moreover, 97 DEGs were detected between stages II and III, of which 68 and 29 genes were upregulated and downregulated, respectively (Figure 2B).

### Figures Functional Classification of DEGs

GO assignments were performed to analyse the functions of these DEGs. These transcripts were assigned to one or more GO terms. In the ovary, GO annotation of the DEGs between the stages I and II showed that both DEGs were categorized mainly into translation and peptide biosynthetic process for biological processes, ribosome for cellular components, and structural constituent of ribosome for molecular functions (Supplementary Figure S1). When stage I and stage III were compared, DEGs were categorized mainly into intracellular signal transduction for biological processes and nucleoside binding for molecular functions (Supplementary Figure S2). Compared to stages II and III, organic acid transport was the most abundant term in biological processes. Cell cortex and sodium:dicarboxylate symporter activity were the top terms in the cellular components and molecular functions, respectively (Supplementary Figure S3). In the hepatopancreas, compared to stages I and II, DEGs were categorized mainly into alcohol metabolic process for biological processes, proteinaceous extracellular matrix for cellular components, and alcohol O-acetyltransferase activity for molecular functions (Supplementary Figure S4). When stage I and stage III were compared, the DEGs were categorized mainly into lipid transport for biological processes, and lipid transporter activity for molecular functions (Supplementary Figure S5). The GO result of stages II and III was similar with that between stages I and III (Supplementary Figure S6).

To further analyze the possible pathways involved in ovarian development and nutritional metabolism, KEGG analysis was performed based on the transcriptome results. The top 16 most enriched metabolic pathways in the ovary and hepatopancreas are shown in Table 3. In the ovary, the top 3 most enriched pathways included phototransduction-fly, phagosome and ECM-receptor interaction. Furthermore, there are many biosynthesis and metabolism enriched pathway of amino acids were obtained in ovary, such as cysteine and methionine metabolism, beta-Alanine metabolism, and phenylalanine metabolism. In the hepatopancreas, DEGs between the stages I and II were significantly clustered in fatty acid biosynthesis and fatty acid metabolism. On comparing stages I and III, riboflavin metabolism, tyrosine metabolism, and metabolic pathways were the most enriched pathways, which were similar results between stages II and III.

**TABLE 3:** Most enriched pathways obtained with KEGG, using the differentially expressed genes from ovary and hepatopancreas of female *E. sinensis*.

Tissue	Pathway	Id	DEGs Number		
			Stage I *vs*. stage II	Stage I *vs*. stage III	Stage II *vs*. stage III
Ovary					
	Phototransduction–fly	dme04745	11	20	5
	Phagosome	dme04145	19	33	9
	ECM-receptor interaction	dme04512	—	—	4
	Hedgehog signaling pathway	dme04340	—	—	5
	Cysteine and methionine metabolism	dme00270	—	—	5
	Ubiquinone and other terpenoid-quinone biosynthesis	dme00130	—	9	3
	beta-Alanine metabolism	dme00410	—	—	4
	Phenylalanine metabolism	dme00360	—	8	3
	Alanine, aspartate and glutamate metabolism	dme00250	—	—	5
	Hippo signaling pathway–fly	dme04391	—	30	—
	Lysine degradation	dme00310	—	—	5
	Taurine and hypotaurine metabolism	dme00430	—	—	2
	Glycosaminoglycan biosynthesis–chondroitin sulfate/dermatan sulfate	dme00532	—	—	2
	Butanoate metabolism	dme00650	—	—	3
	Tyrosine metabolism	dme00350	—	—	3
	Biosynthesis of amino acids	dme01230	—	29	—
Hepatopancreas					
	Fatty acid biosynthesis	dme00061	2	—	—
	Fatty acid metabolism	dme01212	2	—	—
	Riboflavin metabolism	dme00740	1	1	1
	Tyrosine metabolism	dme00350	1	1	1
	Metabolic pathways	dme01100	5	14	7
	Fatty acid degradation	dme00071	1	—	—
	Glycerolipid metabolism	dme00561	1	—	—
	Starch and sucrose metabolism	dme00500	1	3	—
	Ubiquinone and other terpenoid-quinone biosynthesis	dme00130	—	—	1
	Phenylalanine metabolism	dme00360	—	—	1
	Folate biosynthesis	dme00790	—	—	1
	*Glycine*, serine and threonine metabolism	dme00260	—	—	1
	Phototransduction–fly	dme04745	—	2	1
	TGF-beta signaling pathway	dme04350	—	2	1
	FoxO signaling pathway	dme04068	—	2	1
	Hippo signaling pathway–fly	dme04391	—	2	1

### Key Genes Involved in Ovarian Development and Nutrition Metabolism

The genes involved in various processes of ovarian development in the ovary and hepatopancreas are shown in Table 4. Twenty-five key genes were mainly involved in oogenesis, including the ubiquitin-proteasome pathway (UPP), cyclic AMP-protein kinase A (cAMP-PKA) signaling pathway, and mitogen-activated protein kinase (MAPK) signaling pathway. There were four candidate genes for vitellogenesis in the ovary, including *Vg*, *VgR*, vitelline membrane outer layer protein 1 homolog (*VM O 1*) and bone morphogenetic protein 3, of which *Vg* and *VM O 1* were also present in the hepatopancreas. In the ovary, *Vg* expression was downregulated in stages I to II but upregulated in stages II to III. The expression level of *VgR* was the highest in stage II. However, the expression level of *Vg* was upregulated in stages I to III in the hepatopancreas. Nineteen key genes were mainly involved in the process of ovarian development, modulated by a variety of hormones in the ovary and hepatopancreas. Specifically, four genes encode steroid hormones, including estrogen sulfotransferase (*EST*), ecdysone receptor (*EcR*), ecdysteroid-regulated 16 kDa protein and estradiol 17-beta-dehydrogenase 8. Three genes belong to the MF pathway, including 3-hydroxy-3-methylglutaryl-coenzyme A reductase (*HMGR*), juvenile hormone acid O-methyltransferase (*JHAMT*), and mevalonate kinase (*MevK*). The expression levels of *EST* and *EcR* were the highest in ovarian stage III. However, its expression level was the highest in the stage II in the hepatopancreas. The expression levels of estradiol 17-beta-dehydrogenase 8 and ecdysteroid-regulated 16 kDa protein were upregulated from stage I to stage III in the ovary and hepatopancreas. The expression level of prostaglandin reductase 1 was the highest in stage III in the ovary and hepatopancreas. The expression level of hematopoietic prostaglandin D synthase (*H-PGDS*) was the highest in the stage III ovary. The expression level of prostaglandin G/H synthase 2 was the highest in stage II in the hepatopancreas.

**TABLE 4:** Summary of differential and non-differentially expressed genes related to ovarian development in transcriptome of female *E. sinensis*.

Functional category	Gene Id	Gene Name	Tissue	Log2FC		
				Stage II *vs*. stage I	Stage III *vs*. stage I	Stage III *vs*. stage II
Oogenesis						
	evm.model.scaffold215269.3	Ubiquitin carboxyl-terminal hydrolase isozyme L5	O	0.2746	0.5305^a^	0.2607
	evm.model.scaffold189079.103	Ubiquitin domain-containing protein 2	O	0.4189^a^	1.1603^a^	0.7468^a^
	evm.model.scaffold101611.10	Ubiquitin thioesterase OTUB1	O	−0.8314^a^	−1.0975^a^	−0.2624
	evm.model.scaffold5121.29	Ubiquitin−40 S ribosomal protein S27a	O	−0.3673^a^	−0.1518	0.2204
	evm.model.scaffold285327.23	Ubiquitin-conjugating enzyme E2 1	O	0.1338	0.4183^a^	0.2894
	evm.model.scaffold167741.10	Ubiquitin-fold modifier 1	O	0.1310	0.4218^a^	0.2954
	evm.model.scaffold187765.38	Ubiquitin-like protein 5	O	−0.2982	−1.2920^a^	−0.9895^a^
	evm.model.scaffold53153.8	Ubiquitin-protein ligase E3A	O	−0.2168	−0.5410^a^	−0.3194
	evm.model.scaffold122915.10.1	E2 ubiquitin-conjugating enzyme UBE2O	O	−0.0534	−0.5067^a^	−0.4483
	evm.model.scaffold208181.4.1	E3 ubiquitin-protein ligase HUWE1	O	−0.0098	−0.5126^a^	−0.4978^a^
	evm.model.scaffold5267.13	Receptor-type guanylate cyclase Gyc76C	O	0.4804^a^	0.6851^a^	0.2100
	evm.model.scaffold275455.7	26 S proteasome regulatory subunit 7	O	−0.1340	−0.4224^a^	−0.2832
	evm.model.scaffold286953.1	Anaphase-promoting complex subunit 1	O	−0.1821	−0.7939^a^	−0.6068^a^
	evm.model.scaffold90979.2	cAMP-dependent protein kinase type I regulatory subunit	O	0.4616	0.5879^a^	0.1312
	evm.model.scaffold114711.10	Cell division cycle protein 20 homolog	O	0.6218^a^	0.2949	−0.3221
	evm.model.scaffold220627.9	cGMP-dependent protein kinase, isozyme 2	O	−0.5055^a^	−0.9111^a^	−0.3996
	evm.model.scaffold264115.32	Cyclin-dependent kinase 12	O	−0.2223	−0.4166^a^	−0.1891
	evm.model.scaffold167741.6	G2/mitotic-specific cyclin-B3	O	0.0303	−0.4078^a^	−0.4332
	evm.model.scaffold241053.16	Mitogen-activated protein kinase 15	O	−0.4416	−1.1020^a^	−0.6553
	evm.model.scaffold274591.3	Mitogen-activated protein kinase kinase 15	O	−0.2193	−0.5793^a^	−0.3549
	evm.model.scaffold119553.2.1	Protein phosphatase PP2A 55 kDa regulatory subunit	O	−0.2366	−0.4263^a^	−0.1849
	evm.model.scaffold1193895.1	E3 ubiquitin-protein ligase DTX4	H	0.8455	1.7048^a^	0.8544
	evm.model.scaffold205235.1	E3 ubiquitin-protein ligase TRIM9	H	1.5430	2.0905^a^	0.5424
	evm.model.scaffold287175.2	Ubiquitin-conjugating enzyme E2-17 kDa	H	0.5980	1.3555^a^	0.7516
	evm.model.scaffold286953.2	Anaphase-promoting complex subunit 1	H	0.4634	7.3608^a^	6.8887^a^
Vitellogenesis						
	evm.model.scaffold1186140.1	vitellogenin receptor	O	0.1868	0.0315	−0.1500
	evm.model.scaffold243259.1	Vitellogenin	O	−0.8125	−0.6356	0.1771
	evm.model.scaffold154169.14	Vitelline membrane outer layer protein 1 homolog	O	0.7667^a^	1.5412^a^	0.7805
	evm.model.scaffold63979.5	Bone morphogenetic protein 3	O	−1.7986^a^	−1.2749^a^	0.5292
	evm.model.scaffold243259.1	Vitellogenin	H	3.5984	10.8090^a^	7.2030^a^
	evm.model.scaffold154169.14	Vitelline membrane outer layer protein 1 homolog	H	−1.3724	−1.2893^a^	0.0789
Reproduction						
	evm.model.scaffold255843.16	Estrogen sulfotransferase	O	1.6016^a^	2.6699^a^	1.0744
	evm.model.scaffold161675.26	Ecdysone receptor	O	0.1278	0.2139	0.0913
	evm.model.scaffold276475.32	Ecdysteroid-regulated 16 kDa protein	O	0.4953^a^	1.3522^a^	0.8614^a^
	evm.model.scaffold257363.9	Hematopoietic prostaglandin D synthase	O	0.5540^a^	0.9824^a^	0.4333
	evm.model.scaffold295493.8	Prostaglandin reductase 1	O	0.2928	0.5713^a^	0.2838
	evm.model.scaffold179389.8	3-hydroxy-3-methylglutaryl-coenzyme A reductase	O	0.3026	0.1756	0.1222
	evm.model.scaffold79457.3	Juvenile hormone acid O-methyltransferase	O	−0.5923	−0.0881	0.5134
	evm.model.scaffold214315.5	Mevalonate kinase	O	−0.1610	−0.4895^a^	−0.3240
	evm.model.scaffold108751.5	Hydroxysteroid dehydrogenase-like protein 2	O	−0.0682	−0.4897^a^	−0.4164
	evm.model.scaffold99181.11	Insulin receptor	O	0.6917^a^	1.1534^a^	0.4668
	evm.model.scaffold188197.2	Lutropin-choriogonadotropic hormone receptor	O	−0.2798	−1.6597^a^	−1.3748^a^
	evm.model.scaffold282875.53	Molt-inhibiting hormone-like (Fragment)	O	−1.3780	−2.8396^a^	−1.4577
	evm.model.scaffold293791.21	Octopamine receptor beta-3R	O	−0.0333	0.7277^a^	0.7661
	evm.model.scaffold255843.16	Estrogen sulfotransferase	H	3.6768^a^	0.9734	−2.7076
	evm.model.scaffold92161.21	Estradiol 17-beta-dehydrogenase 8	H	0.9808	1.1343^a^	0.1515
	evm.model.scaffold161675.26	Ecdysone receptor	H	0.5430	0.5240	-0.0213
	evm.model.scaffold286593.11	Prostaglandin G/H synthase 2	H	0.1519	−2.4455^a^	−2.6068
	evm.model.scaffold136635.4	Prostaglandin reductase 1	H	−1.2971	2.1285	3.4198^a^
	evm.model.scaffold179389.8	3-hydroxy-3-methylglutaryl-coenzyme A reductase	H	−0.0988	0.4337	0.5285

Log2FC, Log2 Fold changes; O, Ovary; H, Hepatopancreas.

a indicates significant differences among different development stages (*p* < 0.05).

The genes related to nutrition metabolism in the ovary and hepatopancreas are listed in Table 5. There were fifty-six genes related to lipid metabolism in the ovaries and hepatopancreas. Twenty-eight genes involved in lipid catabolism and anabolism were identified in the ovary and hepatopancreas. Among them, 1-acyl-sn-glycerol-3-phosphate acyltransferase 4 (*AGPAT4*), *AGPAT2*, *AGPAT3*, glycerol-3-phosphate acyltransferase 1 (*GPAT1*), *GPAT3*, *GPAT4* and diacylglycerol O-acyltransferase 1 (*DGAT1*) are associated with TAG anabolism. Eight genes involved in lipid oxidation and decomposition were identified in the ovary and hepatopancreas, including acyl-CoA synthetase family member 2 (*ACSF2*), acyl-CoA-binding protein, fatty acid-binding protein (*FABP*) and *FABP homolog 9*. Twenty genes involved in lipid digestion and transportation were identified in the ovary and hepatopancreas. Specifically, in the ovary, eight genes were related to lipid digestion, including phospholipase, monoacylglycerol lipase (*MGL*), hormone-sensitive lipase (*HSL*) and pancreatic triacylglycerol lipase. Three genes were related to lipid transportation were identified: apolipoprotein D, very low-density lipoprotein receptor and low-density lipoprotein receptor 1. In the hepatopancreas, seven genes were related to lipid digestion, such as pancreatic lipase-related protein 2, phospholipase, *MGL* and *HSL*. Moreover, the expression levels of microsomal triglyceride transfer protein (*MTP*) large subunit and low-density lipoprotein receptor-related protein 1 (*LRP1*), which were related to lipid transportation, were upregulated from stage I to stage III in the hepatopancreas.

**TABLE 5:** Summary of differential and non-differentially expressed genes related to nutrition metabolism in transcriptome of female *E. sinensis*.

Functional category	Gene Id	Gene Name	Tissue	FC		
				Stage II *vs*. stage I	Stage III *vs*. stage I	Stage III *vs*. stage II
Catabolism and Anabolism						
	evm.model.scaffold271697.12	Elongation of very long chain fatty acids protein 6	O	0.5674^a^	1.0167^a^	0.4547
	evm.model.scaffold248205.7	Fatty acid synthase	O	−0.0783	−0.7739^a^	−0.6908
	evm.model.scaffold221853.4	Acyl-CoA desaturase	O	1.5896^a^	2.0806^a^	0.4962
	evm.model.scaffold196737.2	Long-chain-fatty-acid-CoA ligase 4	O	0.7121	0.8649^a^	0.1572
	evm.model.scaffold169581.1	Long-chain-fatty-acid-CoA ligase 5	O	−0.8008^a^	−0.9627^a^	−0.1576
	evm.model.scaffold293395.2	Palmitoyl-protein thioesterase 1	O	0.6229^a^	0.4376	−0.1804
	evm.model.scaffold136847.1	Putative fatty acyl-CoA reductase CG5065	O	1.2831^a^	1.3510^a^	0.0725
	evm.model.scaffold283591.6	1-acyl-sn-glycerol-3-phosphate acyltransferase 4	O	0.5443^a^	0.8846^a^	0.3447
	evm.model.scaffold152317.13	1-acyl-sn-glycerol-3-phosphate acyltransferase 2	O	0.0242	0.3818	0.3625
	evm.model.scaffold15989.49.1	1-acyl-sn-glycerol-3-phosphate acyltransferase 3	O	0.3125	0.2474	−0.0596
	evm.model.scaffold285327.35	Glycerol-3-phosphate acyltransferase 3	O	0.0975	−0.2553	-0.3477
	evm.model.scaffold249565.33	Glycerol-3-phosphate acyltransferase 1	O	0.4691	0.9405^a^	0.4765
	evm.model.scaffold271697.41	Glycerol-3-phosphate acyltransferase 4	O	0.4861	0.9647^a^	0.4835
	evm.model.scaffold268161.3	Diacylglycerol O-acyltransferase 1	O	0.6016	0.5050	−0.0917
	evm.model.scaffold282001.14	Phosphatidylserine decarboxylase proenzyme	O	0.0062	0.7179^a^	0.7169
	evm.model.scaffold295607.42	Phosphoethanolamine N-methyltransferase 1	O	0.0338	0.5490^a^	0.5201
	evm.model.scaffold223869.10.1	Long-chain-fatty-acid-CoA ligase	H	−2.6855^a^	−1.3272	1.3569
	evm.model.scaffold248205.7	Fatty acid synthase	H	−5.5894^a^	−1.3584	4.2275
	evm.model.scaffold218373.12	Sterol regulatory element-binding protein 1	H	−1.8143^a^	−0.1608	1.6497
	evm.model.scaffold283591.6	1-acyl-sn-glycerol-3-phosphate acyltransferase 4	H	−0.5452	0.2044	0.7464
	evm.model.scaffold152317.13	1-acyl-sn-glycerol-3-phosphate acyltransferase 2	H	0.1600	0.4702	0.3057
	evm.model.scaffold15989.49.1	1-acyl-sn-glycerol-3-phosphate acyltransferase 3	H	0.1202	−0.0904	−0.2149
	evm.model.scaffold285327.35	Glycerol-3-phosphate acyltransferase 3	H	−0.1026	0.2479	0.3464
	evm.model.scaffold249565.33	Glycerol-3-phosphate acyltransferase 1	H	0.6600	0.2088	0.4557
	evm.model.scaffold271697.41	Glycerol-3-phosphate acyltransferase 4	H	−0.1725	0.0740	0.2448
	evm.model.scaffold268161.3	Diacylglycerol O-acyltransferase 1	H	0.3026	0.3661	0.0588
	evm.model.scaffold282001.14	Phosphatidylserine decarboxylase proenzyme	H	0.4394	2.4321^a^	1.9871^a^
	evm.model.scaffold295607.42	Phosphoethanolamine N-methyltransferase 1	H	−0.5354	0.6836	1.2141^a^
Oxidation and decomposition						
	evm.model.scaffold259733.4	Acyl-CoA synthetase family member 2, mitochondrial	O	0.4573	0.7363^a^	0.2835
	evm.model.scaffold271913.4	Acyl-CoA-binding protein	O	0.1884	0.7673^a^	0.5839^a^
	evm.model.scaffold231737.17	Adiponectin receptor protein	O	0.2763	0.3999^a^	0.1288
	evm.model.scaffold42219.5	Enoyl-CoA delta isomerase 2, mitochondrial	O	0.0461	0.6030^a^	0.5620^a^
	evm.model.scaffold72707.11	Fatty acid-binding protein homolog 9	O	1.1330^a^	2.3843^a^	1.2560^a^
	evm.model.scaffold275455.10	Fatty acid-binding protein, liver	O	0.0325	0.6705^a^	0.7080^a^
	evm.model.scaffold142605.9	NAD-dependent alcohol dehydrogenase	O	0.5327	2.2318^a^	1.7033
	evm.model.scaffold231737.17	Adiponectin receptor protein	H	0.9573	2.1024^a^	1.1387
Digestion and transportation						
	evm.model.scaffold229877.2	Lysosomal acid lipase/cholesteryl ester hydrolase	O	1.1470^a^	1.6044^a^	0.4619
	evm.model.scaffold161691.22	Phospholipase	O	0.5691	0.8682^a^	0.3037
	evm.model.scaffold290469.21	Carboxylesterase 4A	O	0.1542	−1.0236^a^	−1.1728
	evm.model.scaffold264299.1	Cytosolic phospholipase A2	O	1.0282^a^	1.2291^a^	0.2068
	evm.model.scaffold232041.10	Fatty-acid amide hydrolase 2	O	0.6686^a^	0.8983^a^	0.2346
	evm.model.scaffold274729.27	Pancreatic triacylglycerol lipase	O	0.7964^a^	1.0877^a^	0.2955
	evm.model.scaffold292319.39	Monoacylglycerol lipase	O	0.1136	0.5639^a^	0.4553
	evm.model.scaffold232019.28	Hormone-sensitive lipase	O	0.1381	0.1918	0.0592
	evm.model.scaffold48001.8	Apolipoprotein D	O	1.5588^a^	0.2388	−1.3140
	evm.model.scaffold118451.4	Low-density lipoprotein receptor 1	O	0.7358^a^	0.6566^a^	−0.0746
	evm.model.scaffold278801.1	Very low-density lipoprotein receptor	O	3.5713^a^	5.3619^a^	1.7956^a^
	evm.model.scaffold224789.13	Pancreatic lipase-related protein 2	H	2.3436^a^	0.7267	−1.6228
	evm.model.scaffold229877.2	Lysosomal acid lipase/cholesteryl ester hydrolase	H	0.9500	−0.9966	−1.9511^a^
	evm.model.scaffold161691.22	Phospholipase	H	1.7585^a^	−0.0649	−1.8279^a^
	evm.model.scaffold272177.2	Venom carboxylesterase-6	H	−2.0708^a^	−2.1301^a^	−0.0582
	evm.model.scaffold280895.5	Lipase 3	H	−0.7419	−2.6687^a^	−1.9294
	evm.model.scaffold292319.39	Monoacylglycerol lipase	H	0.3879	0.8010	0.4074
	evm.model.scaffold232019.28	Hormone-sensitive lipase	H	0.3352	0.6691	0.3288
	evm.model.scaffold226407.59.4	Microsomal triglyceride transfer protein large subunit	H	0.2871	1.0928^a^	1.3757^a^
	evm.model.scaffold41477.3	Low-density lipoprotein receptor-related protein 1	H	0.6197	1.3408^a^	0.7150

Log2FC, Log2 Fold changes; O, Ovary; H, Hepatopancreas.

a indicates significant differences among different development stages (*p* < 0.05).

### Validation of Gene Expression by Quantitative Real-Time PCR

**FIGURE 3:**
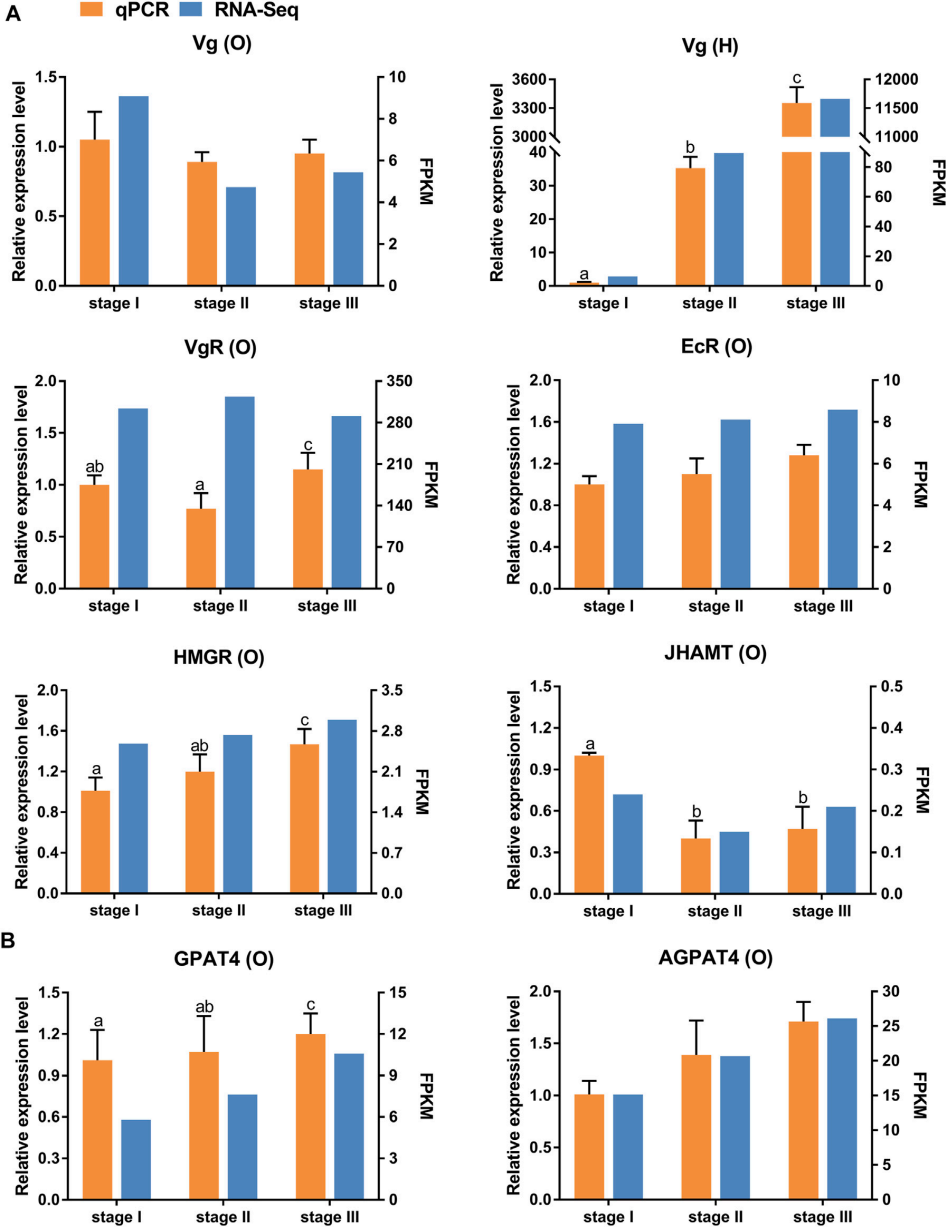
Quantitative expression of eight genes were determined by qPCR in ovary and hepatopancreas from different ovarian development stage of *E. sinensis*. **(A)** genes involved in ovary development. **(B)** genes involved in triacylglycerol metabolism. *Vg*: vitellogenin; *VgR*: vitellogenin receptor; *EcR*: ecdysone receptor; *HMGR*: 3-hydroxy-3-methylglutaryl-coenzyme A reductase; *JHAMT*: juvenile hormone acid O-methyltransferase; *AGPAT4*: 1-acyl-sn-glycerol-3-phosphate acyltransferase 4; *GPAT4*: glycerol-3-phosphate acyltransferase 4. O: ovary; H: hepatopancreas. Different letters on the top of each bar indicate significant differences between the different ovarian stages (*p* < 0.05).

Eight selected genes involved in ovarian development (Figure 3A) and TAG metabolism (Figure 3B) were determined using qRT-PCR to validate the RNA-seq results. The relative expression patterns of these seven genes were consistent with the RNA-seq results. Notably, the qRT-PCR results showed that the expression level of *VgR* was highest in stage III. However, the RNA-seq results showed that the expression level of *VgR* was highest in stage II.

## Discussion

Ovarian development in crustaceans is regulated by several organs and hormones. During the ovarian development of crustacean, protein is involved in many synthetic reactions, such as synthesis of enzymes, hormones and yolk (Wouters et al., 2001). Protein is the first important nutrient for animal growth and reproduction, amino acids are its basic components. Ovarian GO annotation between stages I and II showed the translation and peptide biosynthetic process was main biological processes. The KEGG analysis of the ovary also revealed that many pathways for amino acid synthesis and metabolism were obtained in the ovary, which indicates that the reproduction and growth process in the ovary of this crab need the involvement of proteins and amino acids. In addition, the result of GO annotation showed that lipid transport was the most abundant GO term in the biological processes in the hepatopancreas. And KEGG analysis of the hepatopancreas also showed that DEGs were clustered in the metabolic pathways of various nutrients. This is consistent with role of the hepatopancreas as an absorption and storage organ. As the primary organ of reproductive and nutritional metabolism, the ovary and hepatopancreas play crucial roles during ovarian development (Cheng et al., 1997; Jiang et al., 2009; Nagaraju, 2011; Wang et al., 2018). Furthermore, the conversion from endogenous to exogenous vitellogenesis in crustaceans might be related to hormone regulation (Subramoniam, 2000). Thus, we identified changes in gene expression levels in the ovary and hepatopancreas using transcriptomic analysis during the ovarian development of *E. sinensis*. Both GO and KEGG analyses will facilitate the annotation of these unigenes and investigations of gene function in future studies. These data will provide information to further investigate the molecular regulatory mechanisms involved in oogenesis, vitellogenesis, and nutritional metabolism and transportation in crustaceans.

### Key Genes Related to Ovarian Development

Ovarian formation and maturation are complex processes that require the cooperation of a series of functional pathways (Yang et al., 2015). Oogenesis and vitellogenesis are two finely regulated processes during ovarian maturation in crustaceans (Tsukimura B, 2001). Both mitosis and meiosis are essential for oogenesis. UPP is involved in oocyte meiotic maturation as a key intracellular protein degradation system in the cell cycle (Bebington et al., 2001). In crustaceans, Ubiquitin C-terminal hydrolases L3 (*UCHL3*), *UCHL5*, ubiquitin ribosomal protein S27 (*UbS27*), ubiquitin ribosomal protein L40, and UPP system-related factors participate in the regulation of oogenesis (Han et al., 2010; Wang et al., 2012; Han et al., 2018). In this study, we detected many genes in the pathway that changed in the ovary and hepatopancreas during ovarian development, such as *UbS27a*, ubiquitin-conjugating enzyme, ubiquitin carboxyl-terminal hydrolase isozyme L5, and ubiquitin-protein ligase. Thus, UPP may be involved in oocyte meiosis in *E. sinensis*. Moreover, previous studies have revealed that the cAMP-PKA signaling pathway could regulate the meiosis process of oocytes by activating downstream genes, such as cyclin-dependent kinase 1 (CDK1), cyclin B, anaphase-promoting complex/cyclosome (APC/C) and maturation promoting factor (MPF) (Feng and Thompson, 2018; Firmani et al., 2018). APC/C combines with the cell division cycle protein 20 homolog 1 (Cdh1) and cell division cycle protein 20, which play a vital role in oocyte first meiotic prophase resumption (Marangos et al., 2007). We detected the expression levels of *Cdh1* and anaphase-promoting complex subunit 1 were highest in stage II, which indicated that they were related to the resumption of the first meiotic prophase. The 26 S proteasome hydrolyses cyclin B to maintain the inactive state of MPF, which blocks the oocyte in the diplotene stage of the first meiosis prophase (King et al., 1996; Zachariae and Nasmyth, 1999). In this study, the expression level of 26 S proteasome regulatory subunit 7 was highest in stage 1, which proved that it was related to oocyte first meiotic arrest. In addition, previous studies have indicated that the MAPK signaling pathway participates in the regulation of oocyte meiosis (Gao et al., 2014). In this study, the expression levels of *MAPK 15*, mitogen-activated protein kinase kinase 15, and protein phosphatase PP2A (55 kDa regulatory subunit) belonging to the MAPK signaling pathway were highest in stage I of the ovary, which demonstrated that the MAPK signaling pathway is required in *E. sinensis* to promote oocyte meiosis.

Vitellogenesis is the process of yolk formation via the accumulation Vn and nutrients in oocytes (Chen et al., 2018). Abundant expression of Vg, a Vn precursor, is an indicator of the onset of gonadal maturation in female crustaceans (Tsutsui et al., 2004; Arcos et al., 2011). In crustaceans, the major Vg synthesis site is the ovary in endogenous vitellogenic stage, and the major site of Vg synthesis is the hepatopancreas in exogenous vitellogenic stage (Li et al., 2006). Our study showed that the expression level of ovarian *Vg* was lower than that in the hepatopancreas from stage II to stage III, which indicated that the primary site of *Vg* synthesis changed from the ovary to the hepatopancreas at stage III. In this study, the expression level of *Vg* dramatically increased from stage I to stage III in the hepatopancreas, further supporting the above hypothesis. Similar results have also been reported for other crabs (Zmora et al., 2007; Jia et al., 2013). Moreover, Vg synthesized in the hepatopancreas is immediately transported into developing oocytes by VgR synthesized in the ovary to achieve quick yolk accumulation in oocytes (Bai et al., 2016; Guo et al., 2019). In the present study, the expression level of *VgR* was highest in the stage II transcriptome data. However, the qRT-PCR results showed that the expression level of *VgR* was highest in stage III. Studies by other scholars have suggested that the expression level of *VgR* in stage III was higher than that in stage II during different ovarian developmental stages in *E. sinensis* (Pan et al., 2018; Sun et al., 2020). Therefore, we speculated that this is because the sample of stage III used for transcriptome sequencing was in early-stage III, resulting in not reaching the highest value of *VgR-*mRNA.

The development of crustacean ovaries is controlled by various hormones (Nagaraju, 2011). Estrogen is an important steroid hormone in most crustacean and promotes vitellogenesis and ovarian development (Rodrigue et al., 2002). Previous studies have shown that EST is responsible for the sulfonation and inactivation of estrogens, and plays a crucial role in estrogen homeostasis (Gao et al., 2012). In our study, the *EST* expression level was positively correlated with ovarian stages, indicating that *E. sinensis* may have a self-regulation mechanism to maintain estrogen homeostasis. High levels of ecdysteroids can induce high levels of EcR expression during the ovarian maturation of crustaceans, thereby initiating the ecdysone signaling pathway to be involved in reproduction (Okumura et al., 1992; Subramoniam, 2000). EcR may be involved in vitellogenesis by regulating Vg synthesis (Girish et al., 2015; Gong et al., 2015; Girish et al., 2018). Su et al. (2020) reported that four *EsEcR* isoforms were detected in the ovaries and hepatopancreas in *E. sinensis*. We obtained *EsEcR1* via NCBI BLAST analysis in our study. The *EsEcR1* expression level was concurrently upregulated in the ovary during ovarian development, suggesting that *EsEcR1* may participate in vitellogenesis. MF is a sesquiterpenoid hormone that mediates Vg synthesis in crustacean and is produced by the crustacean MO (Rodriguez et al., 2002; Nagaraju, 2007; Miyakawa et al., 2014). The biosynthesis of crustacean MF is similar to that of insect JH. HMGR is a rate-limiting enzyme in the early steps of JH and MF biosynthesis and is responsible for mevalonic acid biosynthesis (Bellés et al., 2005; Li et al., 2010). The *HMGR* expression was specific to MO (Li et al., 2003; Qiu et al., 2014). In crustaceans, methyl esterification in the final steps of MF biosynthesis is thought to be catalyzed by farnesoic acid O-methyltransferase, and this step is catalyzed by JHAMT in insects (Gunawardene et al., 2002; Holford et al., 2004; Hui et al., 2008; Li et al., 2013). JHAMT plays an essential role in the methyl esterification of fatty acids to MF in crustaceans and is highly specific to MO (Xie et al., 2016). In this study, *HMGR* and *JHAMT* showed low expression levels in the ovary and hepatopancreas. We speculated that the two genes, both highly specific to MO, were difficult to detect in the ovary and hepatopancreas. Furthermore, prostaglandins (PGs) play essential roles in reproductive functions, such as the release of hatching factors and ovarian maturation in crustaceans (Sagi et al., 1995; Reddy et al., 2004). Among them, prostaglandin D2 was synthesized by prostaglandin D synthase (PGDS) (Narumiya et al., 1999). In this study, the expression level of *H-PGDS* was upregulated in the ovary, indicating that it is related to ovarian maturation.

### Key Genes Related to Nutrition Metabolism

Lipids play an important role in the biochemical metabolism and reproduction of decapod crustaceans (Cheng et al., 1997). As a neutral lipid, TAG is the major energy lipid that supports energy needs for reproduction (Yao et al., 2008; Pasquevich et al., 2011). TAG can be synthesized in the ovaries or be transported to the developing ovaries from the hepatopancreas (Alava et al., 2007). Glycerol-3-phosphate acyltransferase (GPAT) is a key enzyme that catalyzes the first step in TAG synthesis (Kennedy, 1961). Four subtypes of GPAT with different functions have been identified in mammals: GPAT1 and GPAT2 participate in TAG synthesis, and GPAT3 is involved in adipocyte differentiation, while GPAT4 plays a pivotal role in mammary lipid droplet (Chen et al., 2008; Gimeno and Cao, 2008; Cattaneo et al., 2012). Zhu et al. (2019) found that two *EsGPATs* subtypes (*EsGPAT1* and *EsGPAT2*), which are more similar to mammalian *GPAT3* and *GPAT4*, exist in *E. sinensis*. In our study, high expression levels of *GPAT1* were found in ovary and hepatopancreas, suggesting that it could participate in TAG synthesis in ovary and hepatopancreas. Although, the expression levels of *GPAT3* and *GPAT4* increased to varying degrees during the ovarian development of *E. sinensis*, its specific function in lipid metabolism requires further investigation. 1-acyl-sn-glycerol-3-phosphate acyltransferase (AGPAT) enzymes catalyze the second acylation step TAG synthesis (Kennedy, 1961). In mammals, several subtypes of AGPAT have exhibited different substrate preferences and physiological functions (Takeuchi and Reue, 2009). In this study, the expression levels of *AGPAT2* and *AGPAT4* were highest in stage III in the ovary and hepatopancreas, whereas the highest expression of *AGPAT3* was found in stage II in the ovary and hepatopancreas. It has been speculated that *AGPATs* may play different roles during oocyte maturation in *E. sinensis*. Diacylglycerol acyltransferase (DGAT) enzymes mediate the last committed step in TAG synthesis by converting diacylglycerol (DAG) to TAG (Harris et al., 2011). In mammals, two DGAT enzymes (DGAT1 and DGAT2) belong to two separate protein families (Coleman and Lee, 2004). In this study, the expression level of ovarian *DGAT1* decreased from stage II to III; however, it increased from stage II to stage III in the hepatopancreas. We speculate that TAGs were mainly synthesized in the hepatopancreas during exogenous vitellogenesis, resulting in decreased ovarian TAG synthesis.

For fatty acid synthesis, fatty acid synthase (FAS) is the key enzyme to endogenous fatty acid synthesis (Chirala and Wakil, 2004). Elongation of very long chain fatty acids (Elovl) is responsible for chain elongation to produce long-chain fatty acids (LCFAs) (Wu et al., 2010). Similar to the study of Sun et al. (2014), the transcription levels of hepatopancreatic *FAS* increased from stage II to III during the ovarian development of *E. sinensis*. These results confirmed that *FAS* is involved in fatty acid synthesis for hepatopancreas-mediated lipid transport during the ovarian development of *E. sinensis*. In our study, *Elovl6* was upregulated in the ovary, demonstrating that *Elovl6* might synthesise LCFAs in the ovary to meet ovarian nutritional requirements. Furthermore, fatty acid-CoA ligase converts free fatty acids into fatty acyl-CoA esters (Cao et al., 2000). The long-chain-fatty-acid-CoA ligase 4 (FACL4) preferentially controls the level of free arachidonic acid (ARA) (Cao et al., 2000). ARA is a precursor of eicosanoids, which plays vital roles in reproduction (Harrison, 1990; Masoudi Asil et al., 2017). In this study, the expression level of ovarian *FACL4* continuously increased from stage I to III, suggesting that it participates in the conversion of ARA in *E. sinensis*. Four genes related to phosphatide metabolism were identified in this study. These results highlight the importance of lipid metabolism during ovarian development in *E. sinensis*.

For lipid oxidation and decomposition, ACSF2 was identified in the ovary of *E. sinensis*. Acyl-CoA synthetase (ACS) would specifically activate fatty acids, essential for β-oxidation (Xu et al., 2012; Gondim et al., 2018). ACSF2 belongs to the ACS family, activating fatty acids by forming a thioester bond with CoA (Yu et al., 2016). In this study, the expression level of *ACSF2* was upregulated from stage I to stage III in the ovary, suggesting that it was related to fatty acids β-oxidation, thereby providing energy during the ovarian development of *E. sinensis*. Furthermore, FABPs transport fatty acids from the cells to various organelles or nuclei for β-oxidation or lipid synthesis (Kaikaus et al., 1990; Jordal et al., 2006). Gong et al. (2010) reported that ovarian *Es-FABP* expression increases with ovarian development in *E. sinensis* to meet the nutritional requirements of ovarian lipids. Coincidentally, the expression level of *FABPs* was also increased in ovarian stage I to III in this study, which further indicated that *FABPs* participate in fatty acid transport during ovarian development of *E. sinensis*.

As mentioned in this study, absorbed nutrients are transported to the ovary from the hepatopancreas during the ovarian development of crustacean (Jiang et al., 2009). In vertebrates, the apolipoprotein (APO) family transports plasma TAGs and cholesterol (Shelness and Ledford, 2005; Wu et al., 2013). MTP transfers intracellular lipid molecules to the APO family, forming lipoprotein particles such as very low-density lipoproteins (Hussain et al., 2003). Lipoprotein receptors can mediate the endocytosis of lipoproteins (Van Hoof et al., 2005). The transfer of APO provides yolk protein precursors and lipid sources to oocytes in the mud crab *Scylla paramamosain* (Zeng et al., 2021). In the present study, the expression level of apolipoprotein D increased from stage I to II in the ovary, suggesting that it might be involved in lipid transportation to facilitate oocyte development. The expression levels of the *MTP* large subunit and *LRP1* consecutively increased from stage I to III in the hepatopancreas. It was speculated that *MTP* and *LRP1* are probably involved in lipid transportation during nutrients production and/or transferred to the oocytes in *E. sinensis*. Lipids, mainly TAG, are taken up and packed into lipid droplets stored in oocytes during ovarian maturation in decapod species (Lee and Walker, 1995). HSL and MGL are intracellular lipases that play a principal role in the hydrolysis of TAG in crustaceans (Miled et al., 2000; Rivera-Perez and Garcia-Carreno, 2011). Specifically, HSL is involved in the hydrolysis of TAG and DAG (Lafontan and Langin, 2009), while MGL hydrolyses monoacylglycerol (MAG) to free fatty acids (Karlsson et al., 1997). In this study, the expression levels of *HSL* and *MGL* were increased from stage I to III in the ovary and hepatopancreas, which indicated that they play an important role in mobilizing energy reserves to promote ovarian development of *E. sinensis*.

## Conclusion

In this study, we performed a comparative transcriptome analysis of the ovaries and hepatopancreas during three vital ovarian stages of *E. sinensis*. The results identified 56 and 43 genes involved in ovarian development and nutritional metabolism, respectively, which are mainly related to the ubiquitin-proteasome pathway, cAMP-PKA signaling pathway, MAPK signaling pathway, triacylglycerol metabolism, and lipid oxidation and transportation. This study will provide new insights into the mechanisms of oogenesis, vitellogenesis, nutritional metabolism and transportation in crustaceans. In the future, the role of these genes and signaling pathways need to be elucidated during the process of crustacean ovarian development and nutritional metabolism.

## Data Availability

The datasets presented in this study can be found in online repositories. The names of the repository/repositories and accession number(s) can be found in the article/Supplementary Material.
