# Supplementary material for: Comparative Transcriptome Analysis Reveals the Process of Ovarian Development and Nutrition Metabolism in Chinese Mitten Crab, Eriocheir Sinensis
Source: Front Genet. 2022 May 24;13:910682. doi: 10.3389/fgene.2022.910682 (PMC9171014; doi:10.3389/fgene.2022.910682)
Supplement: Supplementary file 1 [file DataSheet1.docx]

Supplementary Material

# Supplementary Tables

**Supplementary Table 1**. Specific primers used in this study for quantitative real-time PCR

| **Gene name** | **Primer sequence (5' → 3')** |
| --- | --- |
| Vitellogenin | F: CGCTCCCTGCTGACAAACA  R: TGGGGCGAAATAGGAAAGG |
| Vitellogenin receptor | F: TGGCAACGCCTTCCTTCTGGT  R: GGCACGGTGTTCGCTATCATC |
| Ecdysone receptor | F: AAGGCACGAGACAAGGACAA  R: TGGCTGACATAGGAGACTGAAC |
| 3-hydroxy-3-methylglutaryl-coenzyme A reductase | F: GTGTGGCAGTGATGAAGTCTC  R: AGCAGTGAAGTGAGCAGTGT |
| Juvenile hormone acid O-methyltransferase | F: GCGTGTGCCTTGTGAAGAG  R: TGAGCCACCATGAGTGAGTAG |
| 1-acyl-sn-glycerol-3-phosphate acyltransferase 4 | F: ACACAAGTTCATCATCCGAGAC  R: AAGCACAGCCACCACACT |
| Glycerol-3-phosphate acyltransferase 4 | F: AGGAAGAGGAGGAGGAGGAG  R: CGGTGCGTATGTAGTCAAGAC |
| Fatty acid synthase | F: TCTTCTACGCCATCCATTGCTA  R: TCCTCGCTCTCCAGGTCAA |
| β-actin | F: GCATCCACGAGACCACTTACA  R: CTCCTGCTTGCTGATCCACATC |

# Supplementary Figures


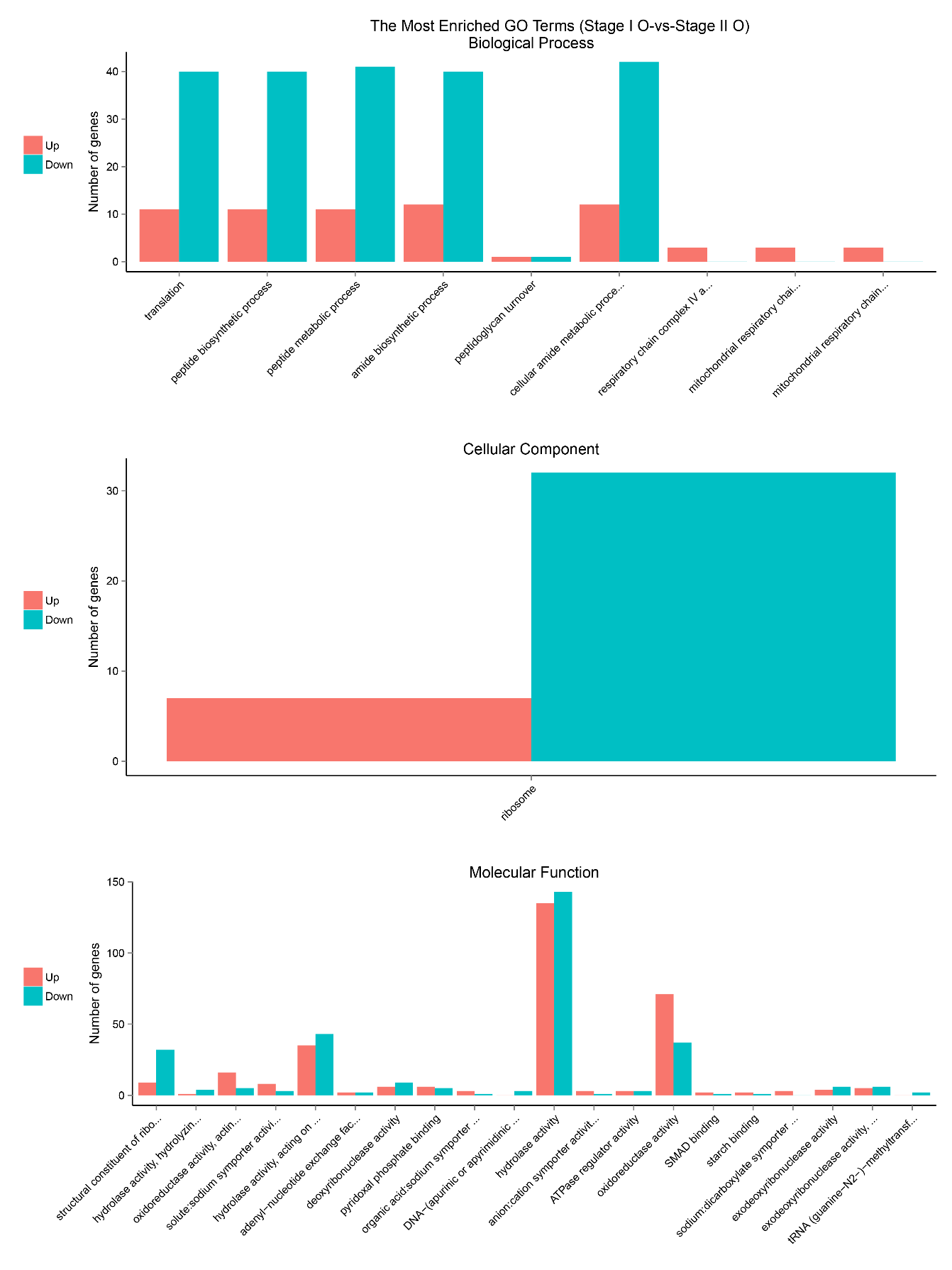
**Supplementary Figure 1.** The most enriched GO terms of differentially expressed genes (DEGs) in ovarian stage I and stage II of *E. sinensis*. O: ovary.


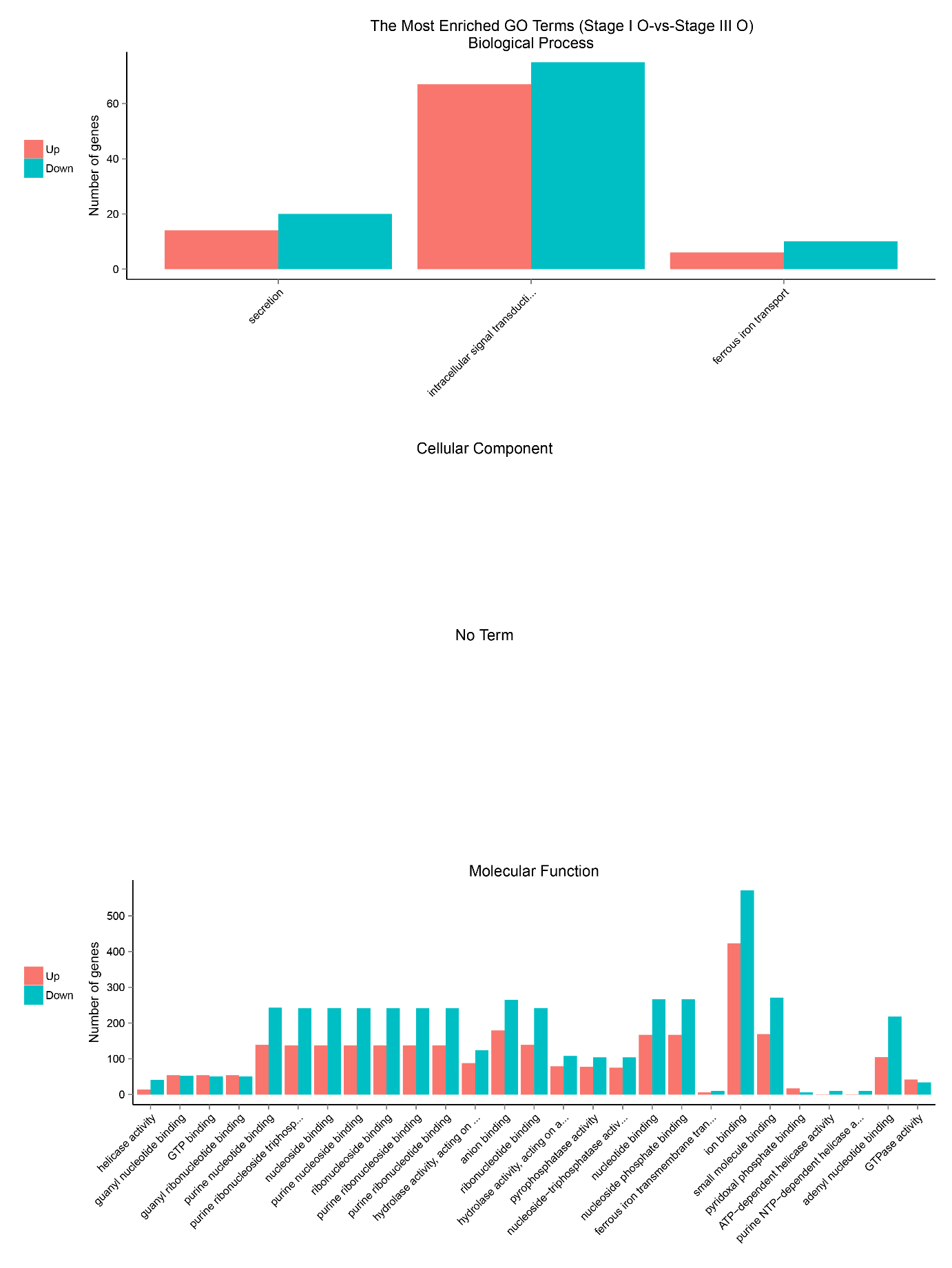


**Supplementary Figure 2.** The most enriched GO terms of differentially expressed genes (DEGs) in ovarian stage I and stage III of *E. sinensis*. O: ovary.


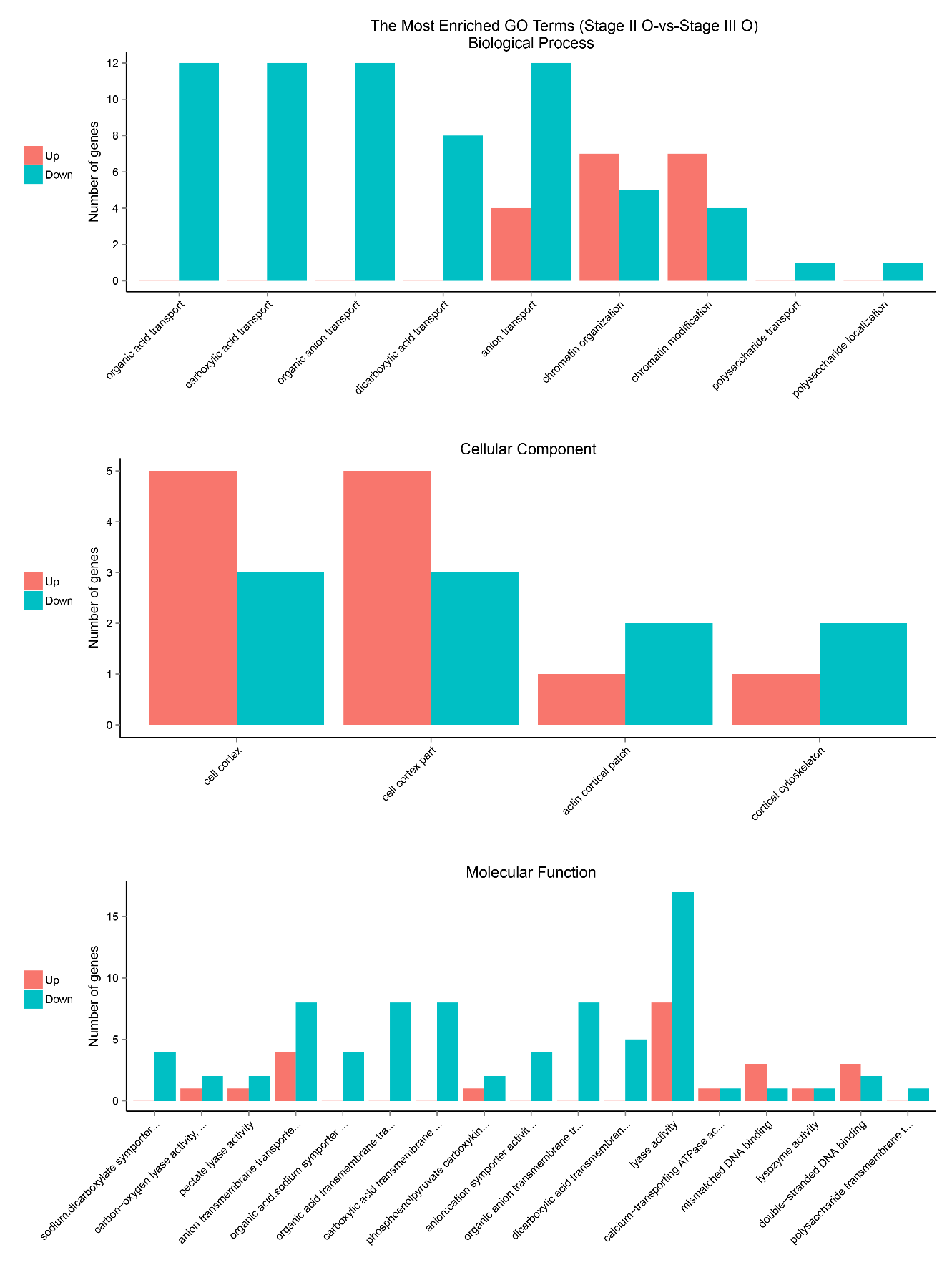


**Supplementary Figure 3.** The most enriched GO terms of differentially expressed genes (DEGs) in ovarian stage II and stage III of *E. sinensis*. O: ovary.


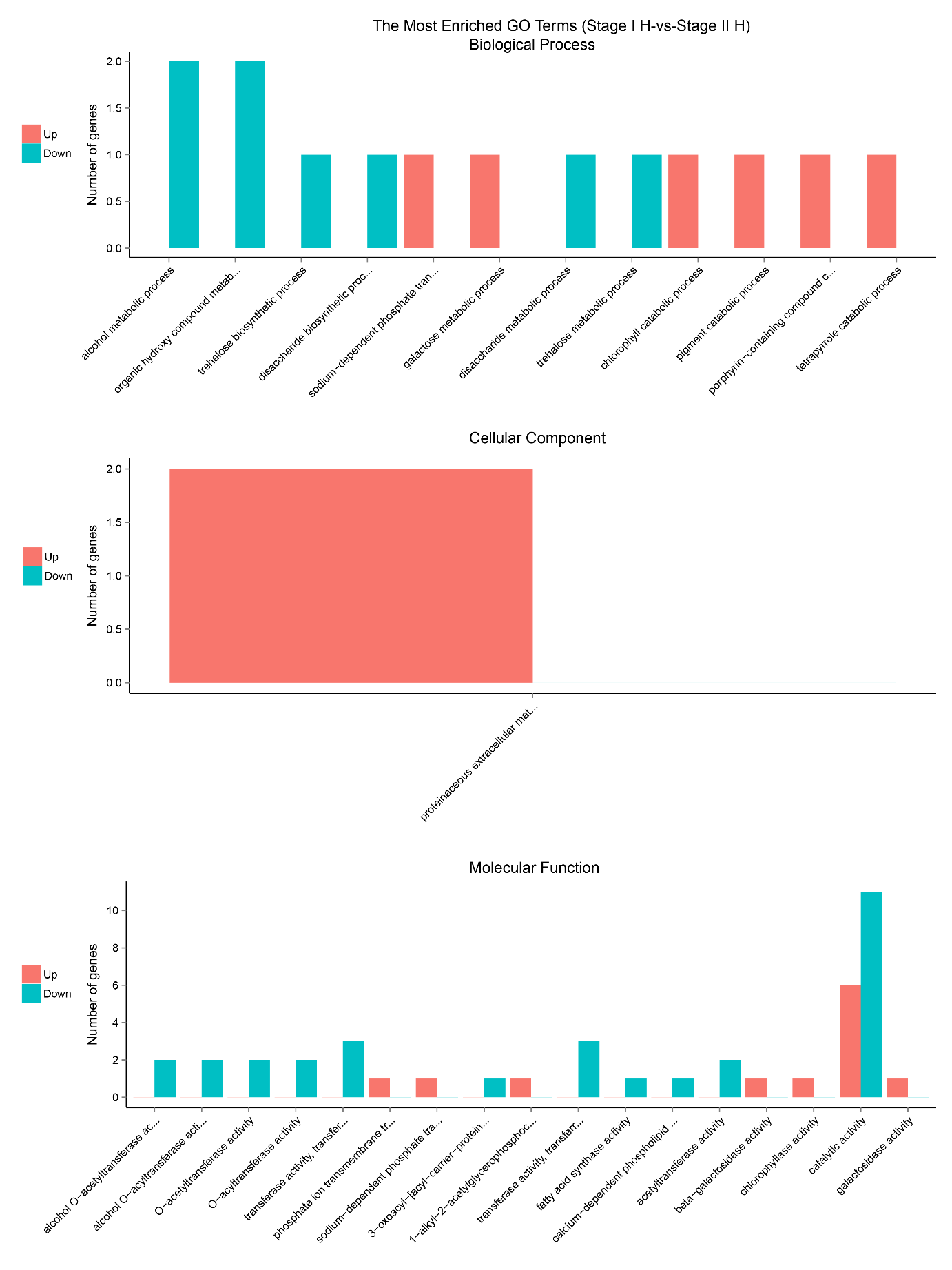


**Supplementary Figure 4.** The most enriched GO terms of differentially expressed genes (DEGs) in hepatopancreatic stage I and stage II of *E. sinensis*. H: hepatopancreas.


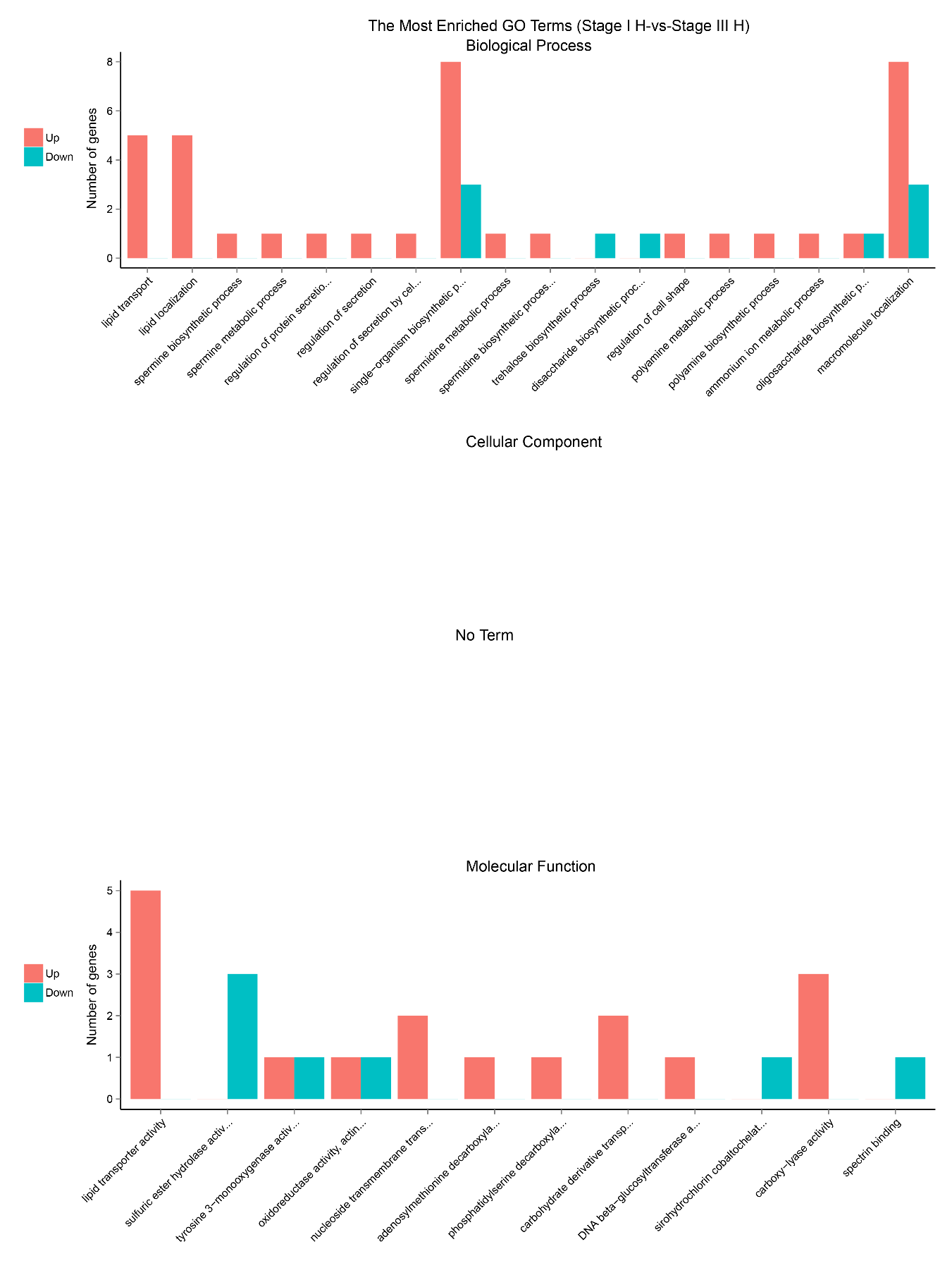


**Supplementary Figure 5.** The most enriched GO terms of differentially expressed genes (DEGs) in hepatopancreatic stage I and stage III of *E. sinensis*. H: hepatopancreas.


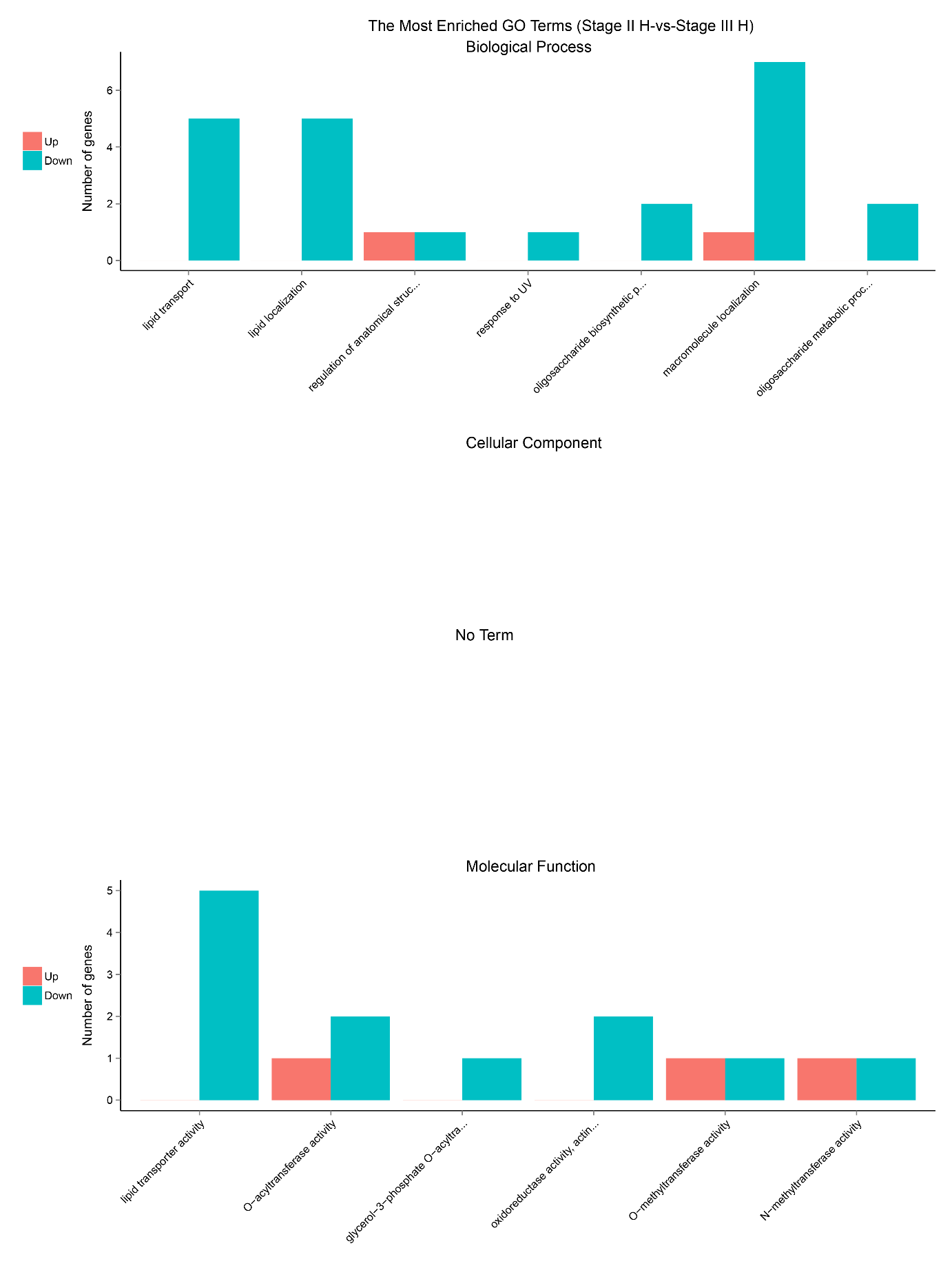


**Supplementary Figure 6.** The most enriched GO terms of differentially expressed genes (DEGs) in hepatopancreatic stage II and stage III of *E. sinensis*. H: hepatopancreas.
